# Supplementary material for: Feasibility of the International Wealth Index and the Gapminder tool as instruments to assess household income and estimate catastrophic expenditure: A prospective patient-level cohort study in India
Source: PLoS One. 2023 May 10;18(5):e0276339. doi: 10.1371/journal.pone.0276339 (PMC10171689; doi:10.1371/journal.pone.0276339)
Supplement: S1 Appendix — (DOCX) [file pone.0276339.s001.docx]

**Appendix**

**Details of the income assessment instruments**

**Supplementary tables and figures**

Supplementary table 1: Details of out-of-pocket payments

Supplementary Figure 1: Scatter plots of self-reported income against the IWI and the Gapminder incomes

Supplementary Figure 2: Q-Q plots of the differences between income assessments

**Full list of PubMed citable co-authors (Crocodile study group collaborators)**

**Details of the income assessment instruments**

**International Wealth Index**

The International Wealth Index is a validated tool for wealth assessment with 12 questions about household assets that individuals might own or not. Each asset has a relative weight and contribution to the IWI score. The final score ranges from 0 to 100 and reflects the household wealth.

| **International Wealth Index** | | |
| --- | --- | --- |
| **Consumer durables** | **Do you own this item?** | **Weight of the item in overall score** |
| Television | Yes/No | 8.612657 |
| Refrigerator | Yes/No | 8.429076 |
| Phone | Yes/No | 7.127699 |
| Car | Yes/No | 4.651382 |
| Bike | Yes/No | 1.84686 |
| **Do you have one or more of these utensils?** Chair, table, clock, watch, water cooker, radio, fan or mixer. | Yes/No | 4.118394 |
| **Do you have one or more of these utensils?** Washer, dryer, computer, motorbike, motorboat, air conditioner, or generator. | Yes/No | 6.507283 |
| **Housing characteristics** | |  |
| Which is your **floor** material? (choose **one** of the options below) | |  |
| Earth, dung, sand | ☐ | -7.558471 |
| Cement, concrete, raw wood | ☐ | 1.227531 |
| Finished floor with parquet, carpet, tiles, ceramic | ☐ | 6.107428 |
| How is your **toilet** facility? (choose one of the options below) | |  |
| Traditional pit latrine, hanging toilet, or no toilet facility | ☐ | -7.439841 |
| Public toilet, improved pit latrine | ☐ | -1.090393 |
| Private flush toilet | ☐ | 8.140637 |
| **How many rooms** are there in your house? (choose **one** of the options below) | |  |
| Zero or one | ☐ | -3.699681 |
| Two | ☐ | 0.38405 |
| Three or more | ☐ | 3.445009 |
| **Public utilities** | |  |
| Access to **electricity** | Yes/No | 8.056664 |
| **Water source:** (choose **one** of the options below) | |  |
| Spring, surface water, unprotected well | ☐ | -6.306477 |
| Public tap, protected well or tanker truck | ☐ | -2.302023 |
| Bottled water or water piped into dwelling or premises | ☐ | 7.952443 |
|  | Constant | 25.00447 |

**Gapminder tool**

The Gapminder Foundation created the Dollar Street project, which aimed to show how people live their daily lives, through pictures taken from all over the world(2). The household income was collected from each household and each picture is matched to the income of the household where it belongs. Reported income, average income for people’s job, extra casual labor, gifted and own-produced food, free housing and assets ownership were used as data sources for household income estimation by the Gapminder team.

Patients were asked to select the household items that look more like their own from the pool of pictures available on the Gapminder website for that particular item. By including all the pictures available on the Dollar Street project for that particular item, we will ensure that a discriminatory range of matching incomes is available. Only pictures from India will be presented to the patients.

The patient income was calculated as a mean average of the incomes matched to the pictures selected by the patient. Please see an example below:

Example of pictures chosen by an hypothetical patient:


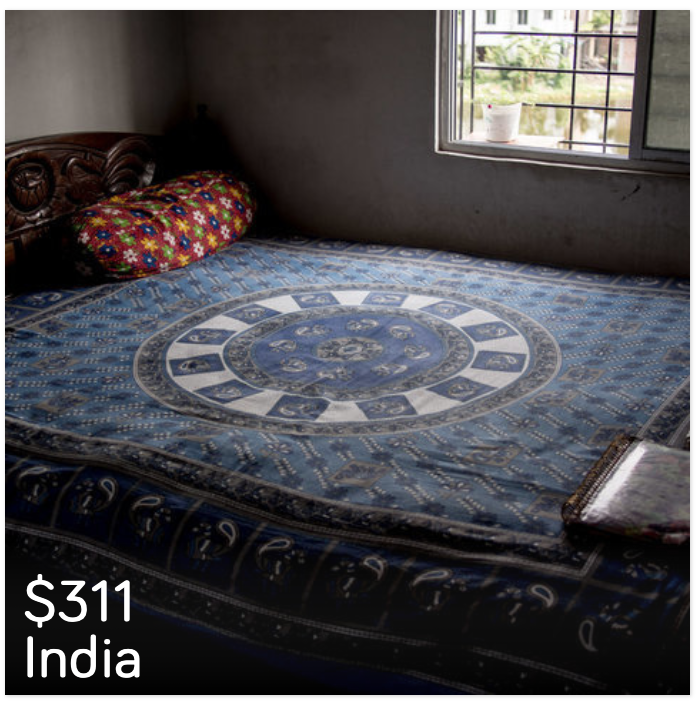

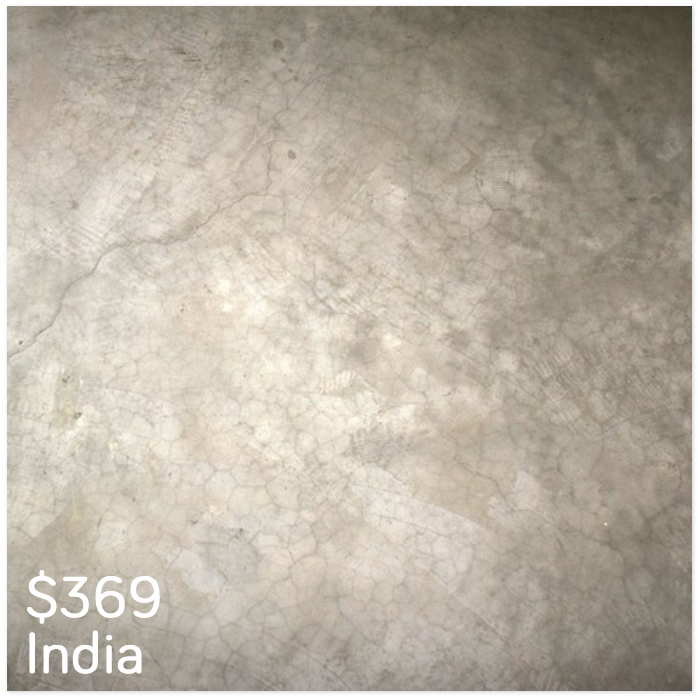

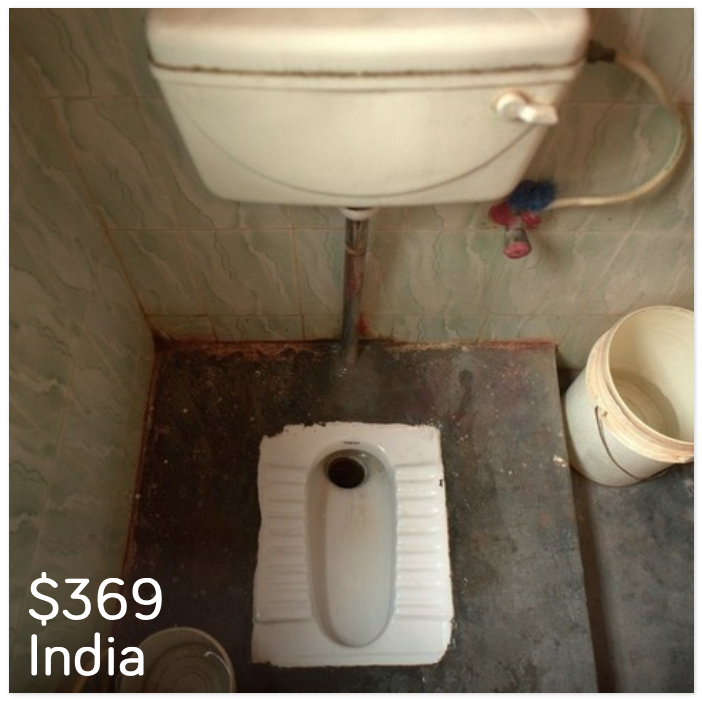

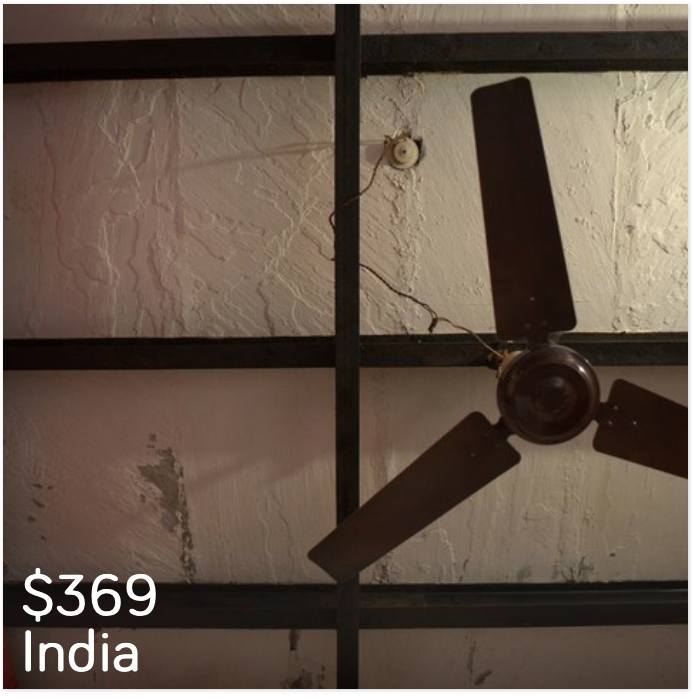

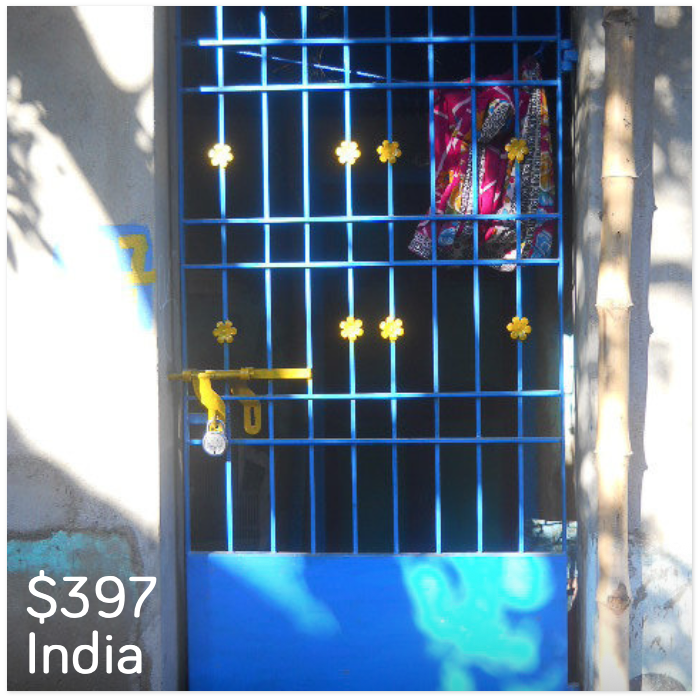


Income calculation = (311+369+369+369+397) / 5 = 363 US dollars (conversion to Indian Rupees explained in the main paper).

**Supplementary table 1: Details of out-of-pocket payments (OOPP)**

| **Type of OOPP for colorectal cancer care** | | **Definition** | **Examples** | **Data source** | **Timing of collection** |
| --- | --- | --- | --- | --- | --- |
| **Direct Costs** | | Out-of-pocket payments made directly by the patient and their household | | | |
| **Direct Medical** | | Out-of-pocket payments for colorectal cancer diagnosis and treatment, made by the patient’s household | | | |
|  | ***In the hospital*** | Direct payments to the recruiting hospital | e.g. surgical fees, radiotherapy fees | Hospital bills | Follow up |
|  | ***Outside the  hospital*** | Direct payments to other hospital or healthcare facility or provider | e.g. drugs from the pharmacy, dressing costs | Patient | First assessment  Follow up |
| **Direct Non-medical** | | Out-of-pocket payments related to the use of cancer care health services in the recruiting hospital, made by the patient’s household | | | |
|  | ***Travelling*** | Payments made by patient’s household for:  - Daily commuting to the hospital (patient and/or companions)  - Long distance travelling (patient and/or household members) | e.g. bus or taxi to the hospital  e.g. flight or train to the hospital | Patient | Follow up |
|  | ***Accommodation*** | Payments made by the patient’s household for accommodation near the hospital in order to access treatment (patient and/or companions) | e.g. staying in a hotel near the hospital to attend multiple outpatient visits | Patient | Follow up |
|  | ***Food*** | Payments made by the patient’s household for food near the hospital in order to access treatment (patient and/or companions) | e.g. food bought by patient’s relatives while staying away from home | Patient | Follow up |
| **Indirect Costs** | | Income losses related work absence due to colorectal cancer care, incurred by the patient (this excludes leave days when salary is not penalised) | e.g. income not earned by the patient during hospital admission | Patient | Follow up |

For the illustrative example on catastrophic expenditure (CE), the out-of-pocket payments at 6 weeks follow-up were used (including direct medical, non-medical and indirect costs). The formula used for catastrophic expenditure (CE) was:

*CE = Out-of-pocket payments > 25% of total household income*

**Supplementary Figure 1: Scatter plots of self-reported income against the IWI and the Gapminder incomes.**

1. **IWI and self-reported income**

**
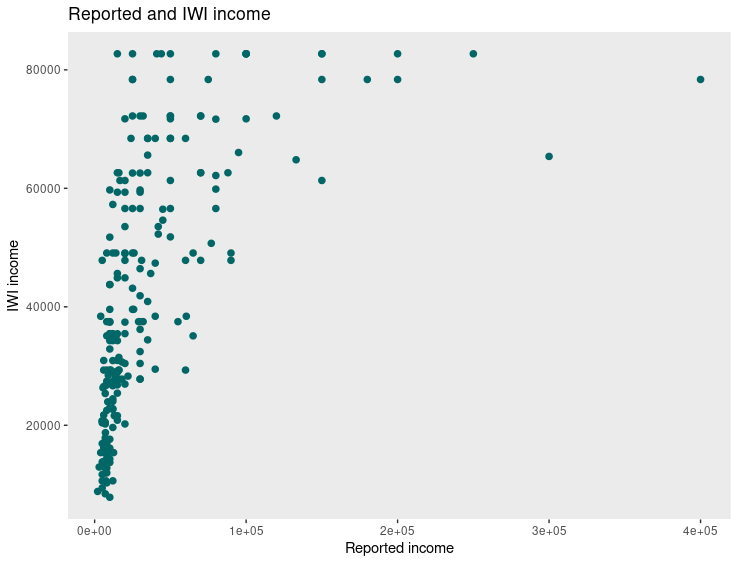
**

1.
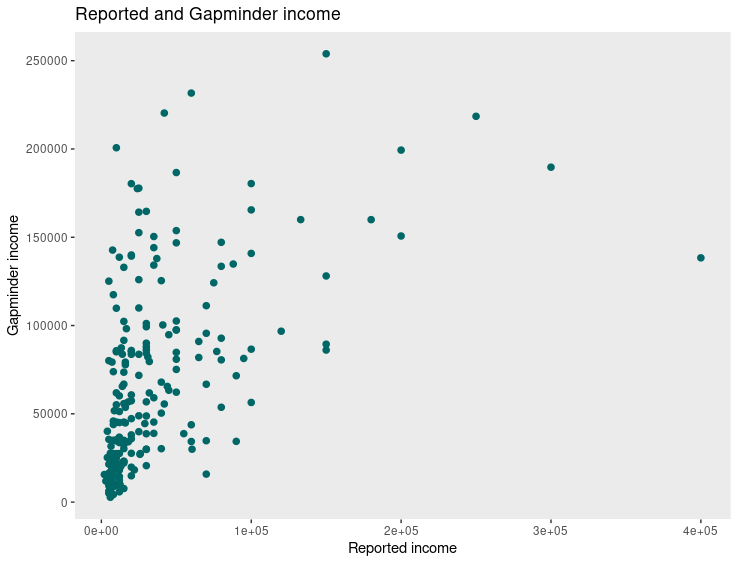
**Gapminder and self-reported income**

**Supplementary Figure 2: Q-Q plots of the differences between income assessments**

1. **Q-Q plot of the difference between the IWI and the self-reported income**

**
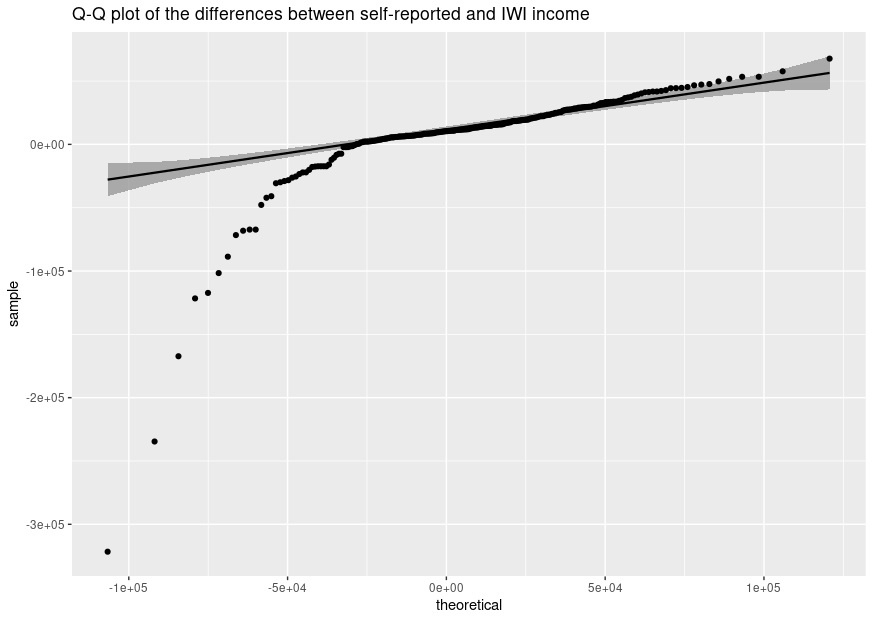
**

1. **Q-Q plot of the difference between the Gapminder and the self-reported income**

**
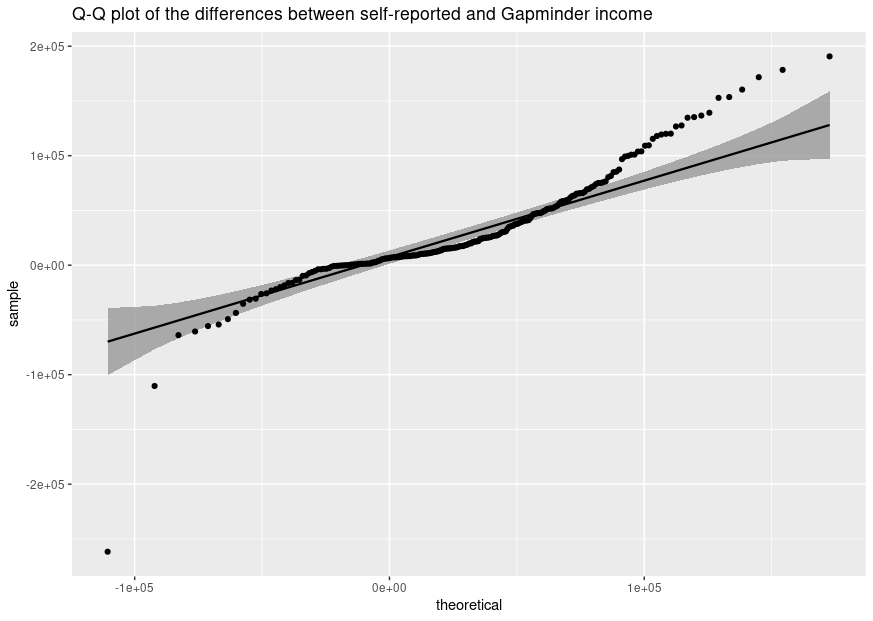
**

**Full list of PubMed citable co-authors (Crocodile study group collaborators)**

Writing group: Bodhisattva Bose^1^, Joanne Clarke^2^, James C Glasbey^3^, Parvez D Haque^4^, Kate Jolly^2^, Pamela A Kingsley^4^, Rohin Mittal^5^, Dion Morton^3^, Raymond Oppong^6^, Ashwin Phillips^4^, Amrit Pipara^7^, Jitendra Rohila^8^, Avanish Saklani^8^, Rajkumar Kottayasamy Seenivasagam^1^, Joana FF Simoes^3^, Atul Suroy^9^, Sreejith K Veetil^4^, Aneel Bhangu^3^, Dhruva Ghosh^4, 9^.

Statistical analysis: Joana FF Simoes^3^, Raymond Oppong^6^, Omar Omar^3^,Aneel Bhangu^3^.

Collaborators (to be completed): *All India Institute of Medical Sciences Rishikesh, Rishikesh, India*: Rajkumar Kottayasamy Seenivasagam*^1^, Bodhisattva Bose^1^, Deepak Sundriyal^1^, Deepa M Joseph^1^, Amoli Tandon^1^, Sunil Kumar Singh^1^, Raunak Verma^1^. *Christian Medical College and Hospital, Ludhiana, India*: Dhruva Ghosh^4^, Parvez D Haque^4^, Pamela A Kingsley*^4^, Ashwin Phillips^4^, Sreejith K Veetil^4^. *Christian Medical College and Hospital, Vellore, India*: Rohin Mittal*^5^, Thomas Samuel Ram^5^, Harish Yezzaji^5^, Mark Ranjan Jesudason^5^, Ashish Singh^5^, David John^5^, Soosan Prasad^5^, Pragnitha Chitteti^5^, Esther Daniel^5^, John Paul^5^, Laura Arthy^5^, *Tata Medical Center, Kolkata, India*: Amrit Pipara*^7^, Robin Thambudorai^7^, Mohandas Mallath^7^, Manas Kumar Roy^7^, Sonia Mathai^7^, Jyotiska Chatterjee^7^, Meenakshi Chakraborty^7^. *Tata Memorial Hospital, Mumbai, India*: Avanish Saklani*^8^, Jitendra Rohila^8^, Ashwin DeSouza^8^, Jayesh Gori^8^, Mufaddal Kazi^8^, Anjali Daphal^8^.

* Denotes principal investigator

Affiliations:

^1^All India Institute of Medical Sciences Rishikesh, Rishikesh, India.

^2^ Institute of Applied Health Research, University of Birmingham, Birmingham, United Kingdom.

^3^ National Institute for Health Research (NIHR) Global Health Research Unit on Global Surgery, University of Birmingham, Birmingham, United Kingdom.

^4^ Christian Medical College and Hospital, Ludhiana, India.

^5^ Christian Medical College and Hospital, Vellore, India.

^6^ Health Economics Unit, Institute of Applied Health Research, University of Birmingham, Birmingham, United Kingdom.

^7^ Tata Medical Center, Kolkata, India.

^8^ Tata Memorial Hospital, Mumbai, India.

^9^ India Hub, NIHR Global Health Research Unit on Global Surgery, Christian Medical College & Hospital, Ludhiana, India.

Corresponding author: Joana FF Simoes, NIHR Global Health Research Unit on Global Surgery, University of Birmingham, UK. ORCID ID: 0000-0002-5294-6282. E-mail: [jfs945@bham.ac.uk](mailto:jfs945@bham.ac.uk).

**References (appendix):**

1. Lindgren M. Gapminder Foundation; 2015 [Available from: <https://drive.google.com/drive/folders/0B9jWD65HiLUnRm5ZNWlMSU5GNEU>.

2. Gapminder Foundation. Dollar Street 2015 [Available from: <https://www.gapminder.org/dollar-street>.
