# Supplementary material for: Feasibility of the International Wealth Index and the Gapminder tool as instruments to assess household income and estimate catastrophic expenditure: A prospective patient-level cohort study in India
Source: PLoS One. 2023 May 10;18(5):e0276339. doi: 10.1371/journal.pone.0276339 (PMC10171689; doi:10.1371/journal.pone.0276339)
Supplement: Attachment — Submitted filename: PONE-D-22-11886 R.pdf [file pone.0276339.s002.pdf]

PLOS ONE

Feasibility of the International Wealth Index and the Gapminder tool as instruments to assess household income: a prospective patient-level cohort study in India

--Manuscript Draft--

**Manuscript Number:**	PONE-D-22-11886
**Article Type:**	Research Article
**Full Title:**	Feasibility of the International Wealth Index and the Gapminder tool as instruments to assess household income: a prospective patient-level cohort study in India
**Short Title:**	International wealth index and Gapminder as indirect tools to assess household income
**Corresponding Author:**	Joana Filipa Ferreira Simoes<br>University of Birmingham<br>Birmingham, UNITED KINGDOM
**Keywords:**	income assessment; catastrophic expenditure; income assessment methods; international wealth index; gapminder.
**Abstract:**	Background: Patient income assessment is required to assess healthcare catastrophic expenditure (Sustainable Development Goal) but self-reported income has several biases. This study aimed to assess the feasibility of the International Wealth Index (IWI) and the Gapminder tool as indirect instruments to assess household income.<br><br>Methods: Prospective cohort study of colorectal cancer patients in five tertiary care hospitals in India (Dec 2020-August 2021). Patient self-reported household income was compared to income estimated from the IWI (twelve questions about household goods) and the Gapminder tool (five pictures of household assets). Agreement between instruments was explored with Bland-Altman methods. Cancer care expenditure from the same cohort was used to illustrate the impact of these tools in catastrophic expenditure rates.<br><br>Results: From the 226 patients included, 99.5% completed the IWI and the Gapminder tool. Overall, self-reported incomes were lower than the estimated from the IWI and Gapminder tools (median incomes: 17350 for self-reported, 37491 for IWI and 51520 for Gapminder). The IWI showed better agreement with the self-reported income than the Gapminder tool. For both instruments, the agreement was better for low income earning households. Illustrative catastrophic expenditure rates range from 71% to 56% to 43% when using self-reported, IWI and Gapminder incomes respectively.<br><br>Discussion: It is feasible to use the IWI and the Gapminder tools to estimate household income although they might overestimate income, with an impact on catastrophic expenditure rates. Further refinement of these tools could enable global monitoring and modelling of catastrophic expenditure from real-world data, at low burden for patients.
**Order of Authors:**	Joana Filipa Ferreira Simoes
	Crocodile study group
**Additional Information:**	
**Question**	**Response**
**Financial Disclosure**<br><br>Enter a financial disclosure statement that describes the sources of funding for the work included in this submission. Review the submission guidelines for detailed requirements. View published research	This work was funded by a National Institute for Health Research (NIHR) Global Health Research Unit Grant (NIHR 16.136.79) using UK aid from the UK Government to support global health research. The funders had no role in study design, data collection, analysis and interpretation, or writing of this report. The views expressed are those of the authors and not necessarily those of the National Health Service, the NIHR, or the UK Department of Health and Social Care.

Powered by Editorial Manager® and ProduXion Manager® from Aries Systems Corporation

articles from *PLOS ONE* for specific examples.<br><br>This statement is required for submission and **will appear in the published article** if the submission is accepted. Please make sure it is accurate.<br><br>**Unfunded studies**<br>Enter: *The author(s) received no specific funding for this work.*<br><br>**Funded studies**<br>Enter a statement with the following details:<br>• Initials of the authors who received each award<br>• Grant numbers awarded to each author<br>• The full name of each funder<br>• URL of each funder website<br>• Did the sponsors or funders play any role in the study design, data collection and analysis, decision to publish, or preparation of the manuscript?<br>• **NO** - Include this sentence at the end of your statement: *The funders had no role in study design, data collection and analysis, decision to publish, or preparation of the manuscript.*<br>• **YES** - Specify the role(s) played.<br><br>* typeset	
**Competing Interests**<br><br>Use the instructions below to enter a competing interest statement for this submission. On behalf of all authors, disclose any competing interests that could be perceived to bias this work—acknowledging all financial support and any other relevant financial or non-financial competing interests.<br><br>This statement is required for submission and **will appear in the published article** if the submission is accepted. Please make sure it is accurate and that any funding sources listed in your Funding Information later in the submission form are also declared in your Financial Disclosure statement.	None.

View published research articles from *PLOS ONE* for specific examples.<br><br>**NO authors have competing interests**<br><br>Enter: *The authors have declared that no competing interests exist.*<br><br>**Authors with competing interests**<br><br>Enter competing interest details beginning with this statement:<br><br>*I have read the journal's policy and the authors of this manuscript have the following competing interests: [insert competing interests here]*<br><br>* typeset	
**Ethics Statement**<br><br>Enter an ethics statement for this submission. This statement is required if the study involved:<br><br>• Human participants<br>• Human specimens or tissue<br>• Vertebrate animals or cephalopods<br>• Vertebrate embryos or tissues<br>• Field research<br><br>Write "N/A" if the submission does not require an ethics statement.<br><br>General guidance is provided below. Consult the submission guidelines for detailed instructions. **Make sure that all information entered here is included in the Methods section of the manuscript.**	Indian Council of Medical Research, University of Birmingham and hospital level ethical approvals were obtained for the study.

Powered by Editorial Manager® and ProduXion Manager® from Aries Systems Corporation

**Format for specific study types**<br><br>**Human Subject Research (involving human participants and/or tissue)**<br>• Give the name of the institutional review board or ethics committee that approved the study<br>• Include the approval number and/or a statement indicating approval of this research<br>• Indicate the form of consent obtained (written/oral) or the reason that consent was not obtained (e.g. the data were analyzed anonymously)<br><br>**Animal Research (involving vertebrate animals, embryos or tissues)**<br>• Provide the name of the Institutional Animal Care and Use Committee (IACUC) or other relevant ethics board that reviewed the study protocol, and indicate whether they approved this research or granted a formal waiver of ethical approval<br>• Include an approval number if one was obtained<br>• If the study involved *non-human primates*, add *additional details* about animal welfare and steps taken to ameliorate suffering<br>• If anesthesia, euthanasia, or any kind of animal sacrifice is part of the study, include briefly which substances and/or methods were applied<br><br>**Field Research**<br><br>Include the following details if this study involves the collection of plant, animal, or other materials from a natural setting:<br>• Field permit number<br>• Name of the institution or relevant body that granted permission	
**Data Availability**<br><br>Authors are required to make all data underlying the findings described fully available, without restriction, and from the time of publication. PLOS allows rare exceptions to address legal and ethical concerns. See the PLOS Data Policy and FAQ for detailed information.	No - some restrictions will apply

Powered by Editorial Manager® and ProduXion Manager® from Aries Systems Corporation

A Data Availability Statement describing where the data can be found is required at submission. Your answers to this question constitute the Data Availability Statement and **will be published in the article**, if accepted.<br><br>**Important:** Stating 'data available on request from the author' is not sufficient. If your data are only available upon request, select 'No' for the first question and explain your exceptional situation in the text box.<br><br>Do the authors confirm that all data underlying the findings described in their manuscript are fully available without restriction?	
**Describe where the data may be found in full sentences. If you are copying our sample text, replace any instances of XXX with the appropriate details.**<br><br>• If the data are **held or will be held in a public repository**, include URLs, accession numbers or DOIs. If this information will only be available after acceptance, indicate this by ticking the box below. For example: *All XXX files are available from the XXX database (accession number(s) XXX, XXX.*).<br>• If the data are all contained **within the manuscript and/or Supporting Information files**, enter the following: *All relevant data are within the manuscript and its Supporting Information files.*<br>• If neither of these applies but you are able to provide **details of access elsewhere**, with or without limitations, please do so. For example:<br><br>*Data cannot be shared publicly because of [XXX]. Data are available from the XXX Institutional Data Access / Ethics Committee (contact via XXX) for researchers who meet the criteria for access to confidential data.*<br><br>*The data underlying the results presented in the study are available from (include the name of the third party*	Data cannot be shared publicly as per national level law in India. Data are available from the NIHR Research Unit on Global Surgery Institutional Data Access / Ethics Committee (contact via jfs945@bham.ac.uk) for researchers who meet the criteria for access to confidential data.<br><br>The data underlying the results presented in the study are available upon request to the corresponding author (jfs945@bham.ac.uk).

Powered by Editorial Manager® and ProduXion Manager® from Aries Systems Corporation

and contact information or URL).<br>• This text is appropriate if the data are owned by a third party and authors do not have permission to share the data.<br><br>* typeset	
Additional data availability information:	

Manuscript Click here to access/download;Manuscript;Income_paper_submission_man

Full title

Feasibility of the International Wealth Index and the Gapminder tool as instruments to assess household income: a prospective patient-level cohort study in India

Short title

International wealth index and Gapminder as indirect tools to assess household income

CROCODILE study group*

**Full list of PubMed citable co-authors in appendix*

Corresponding author: Joana Simoes, NIHR Global Health Research Unit on Global Surgery, Heritage Building, University of Birmingham, Mindelson Way, Birmingham, UK, B15 2TH. Correspondence to: jfs945@bham.ac.uk, ORCID ID: 0000-0002-5294-6282, @JoanaffSimoes, +44(0)7425949987.

Abstract: 250

Word count: 3210

Funding: This work was funded by a National Institute for Health Research (NIHR) Global Health Research Unit Grant (NIHR 16.136.79) using UK aid from the UK Government to support global health research. The funders had no role in study design, data collection, analysis and interpretation, or writing of this report. The views expressed are those of the authors and not necessarily those of the National Health Service, the NIHR, or the UK Department of Health and Social Care.

Conflict of interest / financial disclosure: There are no conflicts to declare.

Abstract

Background: Patient income assessment is required to assess healthcare catastrophic expenditure (Sustainable Development Goal) but self-reported income has several biases. This study aimed to assess the feasibility of the International Wealth Index (IWI) and the Gapminder tool as indirect instruments to assess household income.

Methods: Prospective cohort study of colorectal cancer patients in five tertiary care hospitals in India (Dec 2020-August 2021). Patient self-reported household income was compared to income estimated from the IWI (twelve questions about household goods) and the Gapminder tool (five pictures of household assets). Agreement between instruments was explored with Bland-Altman methods. Cancer care expenditure from the same cohort was used to illustrate the impact of these tools in catastrophic expenditure rates.

Results: From the 226 patients included, 99.5% completed the IWI and the Gapminder tool. Overall, self-reported incomes were lower than the estimated from the IWI and Gapminder tools (median incomes: 17350₹ for self-reported, 37491₹ for IWI and 51520₹ for Gapminder). The IWI showed better agreement with the self-reported income than the Gapminder tool. For both instruments, the agreement was better for low income earning households. Illustrative catastrophic expenditure rates range from 71% to 56% to 43% when using self-reported, IWI and Gapminder incomes respectively.

Discussion: It is feasible to use the IWI and the Gapminder tools to estimate household income although they might overestimate income, with an impact on catastrophic expenditure rates. Further refinement of these tools could enable global monitoring and modelling of catastrophic expenditure from real-world data, at low burden for patients.

Key words: income assessment; catastrophic expenditure; income assessment methods; international wealth index; gapminder.

Introduction

Worldwide, more than 900 million people are experiencing healthcare related catastrophic expenditure[1, 2]. Cancer patients are a particularly vulnerable group and a recent meta-analysis identified that 43% are suffering financial catastrophe due to cancer care[3]. The reduction of catastrophic and impoverishing health expenditure has been prioritised in the Sustainable Development Goals of the World Health Organisation (SCG indicator 3.8.2), to improve access to healthcare and promote and social financial protection for families[4]. Patients are considered to be undergoing catastrophic expenditure if their out-of-pocket payments for healthcare are higher than a proportion of their household income (10 to 25% when using total household income or 40% when using non-subsistence income). The assessment of patient household income is a necessary step to measure catastrophic expenditure rates, monitoring the progress of global health aims and identifying vulnerable populations.

Self-reported income is widely used to assess household income (e.g. household surveys) but is exposed to several biases and limitations. Data from previous studies showed that patients do feel reluctant to answer questions about income and that this can lead to high rates of non-response[5]. Individual characteristics can influence the likelihood of accurate income reporting and surveyed people often have difficulty in interpreting formulated questions about income[6, 7]. Additionally, social desirability bias is commonly present in household surveys responses, introducing over or underestimations depending on the socio-economic and cultural setting, as well as individual circumstances at the point of data collection[8, 9]. These limitations of self-reported income can have an impact on population-level estimations (e.g. poverty lines) and policy decision making at national and global level[10, 11].

To the best of our knowledge, direct income reporting is the standard method to assess income and there are no alternatives. This study aimed to assess the feasibility of the International Wealth Index (IWI) and the Gapminder tool as indirect measures of household income, in a cohort of patients with colorectal cancer in India.

Material and methods

Study design and setting

This is a prospective multicentric cohort study conducted in tertiary care hospitals in India, including consecutive patients with a new treatment decision for colorectal cancer from December 2020 to August 2021. The overall goal of this cohort study was to determine catastrophic expenditure and treatment adherence rates among colorectal cancer patients in India. In order to assess catastrophic expenditure, both out-of-pocket payments for cancer and patient household income assessment were collected. This is a pre-planned analysis comparing three different instruments of assessing patient household income, to assess the feasibility of their use.

The study protocol has been published and the study is registered on ClinicalTrials.gov (NCT04517032) and with the Central Trials Registry of India (CTRI/2020/09/027896) [12]. Indian Council of Medical Research, University of Birmingham and hospital level ethical approvals were obtained for the study. The hospitals started data collection as soon as their ethical approval was obtained, recruiting a maximum of seventy patients per hospital until the total sample size was achieved.

Patient inclusion and data collection

All consecutive patients with a new treatment decision for colorectal cancer were identified from multidisciplinary team meetings or outpatient clinics, depending on local pathways. Recruitment and baseline data collection were conducted in the first hospital visit after patient identification, during which patients completed a self-reported income assessment as well as the International Wealth Index and the Gapminder tool.

Further data variables were collected: age, sex, education level (primary, secondary, graduate, post-graduate, did not attend school), job skill level (categorised according to the International Standard Classification of Occupations [13] as high, medium, low or not specified), distance from the patients' house to the hospital and number of people living in the household (number of adults and children sharing the same house). Tumour and treatment plan details were also collected at the point of patient

inclusion: tumour location (colon or rectum), tumour stage (local or advanced, as per AJCC 8th edition Tumour Node Metastasis classification) and treatment intent (curative or palliative). Follow-up data was collected at 6 weeks, 3 months and 6 months after the baseline assessment, including details of the treatment course and payments made by patients for cancer care (direct medical, non-medical and indirect payments are fully described in Supplementary Table 1).

Income assessment instruments

Self-reported income

Patients reported the total amount of money earned monthly by all the household members who work and produce income, in Indian Rupees (INR, ₹). This could include a regular monthly income in the form of a salary or an average monthly income as a result of a variable daily wage.

International Wealth Index

The International Wealth Index (IWI) is a twelve-point questionnaire where patients reported whether they owned a number of consumer durables (television, refrigerator, phone, car, bicycle, cheap utensils e.g. chair and expensive utensils e.g. air conditioner) and described their household characteristics (floor material, toilet facility, number of rooms, access to electricity and water source)[14]. Based on the responses to these questions, a final score is calculated, ranging from 0 to 100 where a low score relates to lower wealth levels and a high score relates to a wealthier household. The full score is available in the Appendix: Details of the income assessment instruments.

.

The final IWI score was translated into an income figure using a previously available formula that explains the relationship between income and the IWI score in 2015 United States Dollars (USD)[15]. The following formula was obtained by computing the IWI in multiple population based households (including the India Human Development Survey).

*IWI income (per day, in USD) = 1.489056*exp(0.02918)*

In order to obtain a figure that is comparable to the reported income in 2021 Indian Rupees, the IWI income in 2015 United States Dollars (USD) was multiplied by purchase power parities (PPP) for USD-INR exchange and by the consumer price index to account for inflation between 2015 and 2021[16].

Gapminder tool

The Gapminder Foundation has created the Dollar Street project where a pool of pictures of household and personal goods are available and matched to an income reported by the household members to the Gapminder team [17]. The patients were shown five categories of pictures from Indian households (all the available at the time of study design): houses, kitchens, floors, toilets and beds. The patient was invited to select the house, kitchen, floor, toilet and bed that looked more similar to the one their own, to which an income was then matched (more details are available in the Appendix: Details of the income assessment instruments). The mean average of the income matched to the selected pictures was obtained and converted from USD to INR using purchase power parities, as follows:

$$\text{Gapminder income} = \frac{(\text{Income matched to house} + \text{kitchen} + \text{floor} + \text{toilet} + \text{bed})}{5} * \text{PPP}$$

Statistical analysis and data handling

Continuous variables were described with standard summary metrics: mean and standard deviation when normally distributed and median and inter-quartile range (IQR) when skewedly distributed. Categorical variables were described using frequency tables. A Bland-Altman analysis was performed to describe the agreement between (1) self-reported and IWI income and (2) self-reported and Gapminder income. In the Bland-Altman plots, the difference between reported and estimated incomes was plotted against the mean average of those the same incomes[18]. A standard Bland-Altman analysis with agreement limits based on 95% confidence intervals for the observations was not possible, provided that the income data did not comply with normality assumptions. We opted for the description of the proportion of patients for whom the differences between reported and estimated incomes were within a particular range (methodology suggested by Bland and Altman for non-

parametric data[19]. This allows an understanding of the range of differences between reported and estimated incomes, as well as the description of any patterns in the Bland-Altman plots. Missing data was described for all variables in the tables and plots.

Illustration of the impact of income assessments in catastrophic expenditure rates

Cancer care related expenditure was collected from the same cohort of patients, at 6 weeks after a new decision for treatment by the clinical team in charge of the patient (costs were assessed at other timepoints and will be published separately). This was used to illustrate the impact of the different income assessment tools in catastrophic expenditure rates. Catastrophic expenditure was defined as out-of-pocket payments for cancer care being 25% of total household income for the purpose of this analysis, as per the most updated WHO definition of the Sustainable Development Goals [4]. Full description of the out-of-pocket payments included in this illustration are available in Supplementary Table 1.

Results

Patient characteristics

In total, 226 patients were included from five tertiary care hospitals in India. The median age was 52 years and 63.3% (143/226) of the patients were men. The household size was of one to three people in 26.5% of the patients (60/226) and four to six people in 52.2% of the patients (118/226). Overall, 104 of all patients did not have a job (being retired or housewives), with the remaining patients having a similar distribution in terms of professional skill levels (low 13.3%, medium 17.7% and high 17.7%). Regarding the education levels, the majority of the patients completed primary (22,6%), secondary (36.7%) or graduate degrees (27.9%), with a minority not having attended school (4.4%). Regarding clinical features, 118 patients had rectal cancer and 108 had colon cancer. Most of the patients had a treatment plan with a curative intent (83.1% [187/226]) and 16.9% received palliative treatment only. For full patient characteristics see Table 1.

Self-reported, IWI and Gapminder income distribution

From the 226 patients included, 99.5% completed all the three instruments of income assessment. Only one patient did not complete the IWI and another patient did to respond to the Gapminder tool. Overall, the median reported income (17350₹, (IQR 10000-40000)) was lower than median income assessed using the IWI (37491₹, (IQR 25421-61322)) and from the Gapminder tool (51520₹, (IQR 22120-91128)). The distribution and metrics of the self-reported IWI and Gapminder incomes are shown in Figure 1.

The distribution of the self-reported incomes was more skewed to the left (lower incomes), meaning that more patients reported a lower income directly, when compared to the incomes estimated with the IWI and the Gapminder tool (Figure 2). The distribution of IWI income figures is more homogeneous, and the figures are contained within a narrower range, when compared to the Gapminder or the self-reported income where more outliers exist. The Gapminder income follows a similar distribution when compared to the reported income but with a flattened density curve, where the results are not as skewed towards low incomes as they are for self-reported income.

Supplementary Figure 1 (A) and (B) shows scatter plots exploring the association between the self-reported income and estimated incomes using the IWI and the Gapminder tool respectively. Both estimated incomes show a positive non-linear association with self-reported incomes. The association is strong for low reported incomes, becoming weaker as the reported income increases.

Agreement between self-reported, IWI and Gapminder incomes

The differences between reported and estimated incomes show lower dispersion for the IWI, compared to the Gapminder income (Figure 3A). The proportion of patients for whom the estimated income is within 50000 INR of the self-reported income is 92.8% for the IWI and 70.2% for the Gapminder tool. Similar trends are observed for differences within lower limits (25000 INR) and higher limits (100000 INR), reinforcing better agreement for the IWI (see Figure 3B).

In the Bland-Altman plots describing agreement for both the IWI and the Gapminder tool (Figure 3A), there is a funnel shaped distribution of the differences between self-reported and estimated incomes. The differences are closer to zero for low income earning households and farther from zero as the household income increases, demonstrating a worse degree of agreement for high income earning households. Standard 95% confidence interval for agreement can't be drawn for this data as the differences between income assessments didn't follow a normal distribution (Q-Q plots shown in Supplementary Figure 2).

Illustration of catastrophic expenditure rates

Within six weeks of having received a new decision for treatment, this cohort of patients had a median expenditure of 202291INR for cancer care (interquartile range: 107900 - 308010 INR). The rates of catastrophic expenditure calculated using self-reported and estimated incomes were different (Figure 4). The rate of catastrophic expenditure for this cohort of patients was highest when the self-reported income was used (80.5%), when compared to the rates obtained with the IWI income (73.5%) or the Gapminder tool (56.7%).

Discussion

The IWI and the Gapminder tool are feasible and promising instruments to assess household income in global health research studies. The vast majority of this cohort of colorectal patients from India was able to complete both instruments. When using the IWI index to estimate household income, 92.8% of the patients will have an estimated income that differs up to 50000 INR from their self-reported income. The Gapminder tool shows worse agreement, with 88.9% of the patients having an estimated income that differs up to 100000 INR from the self-reported income. The use of different tools to assess income resulted in different catastrophic expenditure rates within the cohort, highlighting that further research and refinement of methods is needed to monitor this well-established Sustainable Development Goal at global scale.

The fact that self-reported incomes are generally lower than estimated incomes is unsurprising. Firstly, patients might feel uncomfortable revealing their income due to social pressures[8, 9, 11]. Secondly, all income data were collected at the point of inclusion in the cohort study which was, by definition, coincident with a new treatment decision for colorectal cancer. At this point in time, many patients could have been seeking funds from the hospital and the government to pay for their cancer care. Given that government and charity funds are usually allocated to patients based on their earnings, this might have played a role in patients underreporting their income[20]. Thirdly, the self-reported income was collected from household members wages and did not include any other sources of income (e.g. inheritance). Even though self-reported incomes tended to be lower, the most severe outlying income figures were actually self-reported. This can reflect the inability of the indirect tools to capture these incomes, but we can’t exclude a previously described form of social desirability bias where some patients felt like they should overreport their incomes [11].

The International Wealth Index and the Gapminder tool were both designed to allow a comparison of wealth levels across households[14, 17]. It is expected that households generate wealth through their income but also through other sources of purchasing power. There is no consensus on the best definition and threshold for

catastrophic expenditure and some authors argue that wealth and expenditure levels might be a better reflection of households' capacity to pay for cancer care[21]. The indirect tools have the advantage of assessing wealth more holistically, which might justify why IWI and Gapminder incomes being higher than the self-reported income. Additional factors can contribute to higher income figures with the IWI and the Gapminder tool, such as patients owning donated household goods that typically belong to higher income households or the personal choice to rent versus buy a house (renting requires less short-term investment for a better house).

The authors of the IWI have described that a truncation effect exists in this index, meaning that it has less discriminative power in very low and very high levels of wealth, as the score is limited to the number and value of the included assets[14]. This explains why the income figures obtained with the use of the IWI showed a narrower distribution. This can also explain why the agreement between the IWI and the self-reported income is worse for high income earners.

The Gapminder tool showed a less skewed distribution of incomes than self-reporting or the IWI, with more patients having higher incomes. This might reflect a better ability to discriminate wealth but can also be a result of this tool including multiple sources of data to match a household to an income (e.g. minimum and average wages, own food production, average consumption for specific groups of the population)[15]. The way that this tool was designed and used in the study could have contributed to worse agreement with self-reported income when compared to the IWI. Although there was a wide range of house items for the patient to choose from (around 30 items per category), patients could have struggled to find items that look like their own. We also acknowledge that similar pictures could be matched to quite different incomes, e.g. private flush toilets looking quite similarly were owned by households living on 1295 USD a month and 3477 USD a month, potentially resulting in very different income estimations.

Although this study is a unique evaluation of three different methods to assess household income to date, some limitations must be acknowledged. Firstly, although the completion rates were very high, full limitations of the use of the IWI and Gapminder tools were not explored in this study (e.g. biases and barriers to

respond). Secondly, although this is a multicentric cohort study, it only included tertiary care hospitals in India, limiting generalisability for other countries and other levels of care in India. Finally, the IWI and the Gapminder tools revealed better agreement for low-income earning households, compromising the applicability of the results to high-income patients.

Future research should focus on the refinement of these tools, enabling their use worldwide. From our data, we can conclude that the IWI shows better agreement with self-reported incomes, but high income earning households are underrepresented. The IWI was derived from a set of household surveys from which several items were selected and their association to household income was drawn into a weighted score. A refinement of this index to capture some items typically owned by wealthy households could improve its discriminative power for high-income earners and its overall generalisability. Regarding the Gapminder tool, the estimated incomes follow a distribution shape comparable to the self-reported income but are more dispersed. For future studies using this tool, a selection of fewer pictures for each category (e.g. ten kitchens representative of ten different income levels), representative of well-defined income levels can improve its ability to estimate accurate income figures.

A qualitative assessment of the use of these tools is necessary to achieve a better understanding of their strengths, limitations and potential biases. A consensus process involving patients, researchers and decision makers might be needed ahead of its global use, enabling the development of a tool that matches goals at individual and community levels.

Provided the necessary refinements, these tools have the potential to facilitate real-world assessment and monitoring of healthcare related catastrophic expenditure, without the need for direct income assessment. Moreover, data on house characteristics and household goods is routinely collected in several household surveys, meaning that the IWI and the Gapminder tool could allow large scale modelling of catastrophic expenditure rates from population survey data. Although more research is needed, the high completion rates and the apparent low complexity

of both the IWI and the Gapminder tool allows us to conclude that they are promising tools in global health research

Conclusions

This study demonstrates that household income assessment can be performed with indirect tools, avoiding self-reported income. Although these alternative tools need refinement, they are promising assets for monitoring and modelling of catastrophic at global scale.

References

1. World Health Organization and the International Bank for Reconstruction and Development / The World Bank, Global monitoring report on financial protection in health 2021. 2021.
2. Wagstaff A, Flores G, Hsu J, Smitz MF, Chepynoga K, Buisman LR, van Wilgenburg K, Eozenou P, Progress on catastrophic health spending in 133 countries: a retrospective observational study. Lancet Glob Health, 2018. Feb;6(2):e169-e179.
3. Doshmangir L, Hasanpoor E, Abou Jaoude GJ et al,, Incidence of Catastrophic Health Expenditure and Its Determinants in Cancer Patients: A Systematic Review and Meta-analysis. Appl Health Econ Health Policy. 2021. 19, 839–855.
4. Global Health Observatory, W.H.O. Accessed 24.02.2022; Available from: https://www.who.int/data/gho/data/themes/topics/indicator-groups/indicator-group-details/GHO/incidence-of-catastrophic-health-spending-sdg-indicator-3-8-2-.
5. Moore JC, Loomis L, Reducing Income Nonresponse in a Topic-Based Interview. RESEARCH REPORT SERIES, 2022.
6. Angel S, Heuberger R, Lamei N,, Differences Between Household Income from Surveys and Registers and How These Affect the Poverty Headcount: Evidence from the Austrian SILC. Soc Indic Res 138, 575–603 2018.
7. Moore JC, Stinson L, Welniak EJ,, Income measurement error in surveys: a review. Journal of Official Statistics, 2000. 16, 4, 331-361.
8. Fisher RJ, Katz J., Social-desirability bias and the validity of self-reported values. Psychology & Marketing, 1999. 17:2 105-120.
9. TJ, DeMaio, Survey desirability and survey measurement: a review, in Surveying subjective phenomena 2. 1984, Russel SAGE Foundation New York.
10. Czajka JL, Danmead G, Income Data for Policy Analysis: A Comparative Assessment of Eight Surveys 2008, Office of the Assistant Secretary for Planning and Evaluation (ASPE), Department of Health and Human Services (HHS).
11. Hariri JG, Lassem D, Income and outcomes: Social desirability bias distorts measurements of the relationship between income and political behavior. Public Opinion Quarterly, 2017. 81:2, 564–576.

12. CROCODILE study group, Catastrophic expenditure rates and barriers for treatment adherence in patients with colorectal cancer in India: The CROCODILE study protocol. Colorectal Disease, 2021. 23(8):2161-2172.
13. International Labour Office Geneva, International Standard Classification of Occupations (ISCO-08). 2012: ISBN 978-92-2-125953-4.
14. Smits J, Steendijk R., The International Wealth Index (IWI). Soc Indic Res, 2015. 122, 65–85.
15. Lindgren M, Detailed income calculations for Dollar Street. 2015 01.02.2022]; Available from: https://drive.google.com/drive/folders/0B9jWD65HiLUnRm5ZNWlMSU5GNEU.
16. Organisation for Economic Co-operation and Development (OECD). Purchasing power parities (PPP) (indicator). 2022, Accessed 22.01.2022; Available from: https://data.oecd.org/conversion/purchasing-power-parities-ppp.htm.
17. Foundation, G. Dollar Street Project. 2015, Accessed 01.05.2020; Available from: https://www.gapminder.org/dollar-street?countries=in.
18. Bland JM, Altman D., Statistical methods for assessing agreement between two methods of clinical measurement. The Lancet, 1986. 327, 8476, 307-310.
19. Bland JM, Altman D., Measuring agreement in method comparison studies Statistical Methods in Medical Research, 1999. 8:2 135-160.
20. Pramesh CS, BadweR., Borthakur BB, et al, Delivery of affordable and equitable cancer care in India. The Lancet Oncology,, 2014. 15:6, 223-233.
21. Jan S, Laba T., Essue BM, et al,, Action to address the household economic burden of non-communicable diseases,. The Lancet, 2018. 391:10134, 2047-2058.

Figure 1 Click here to access/download;Figure;Figure 1.pdf

Figure 1: Distribution and summary metrics of patient household monthly income, assessed by self-reporting, the International Wealth Index and the Gapminder tool.

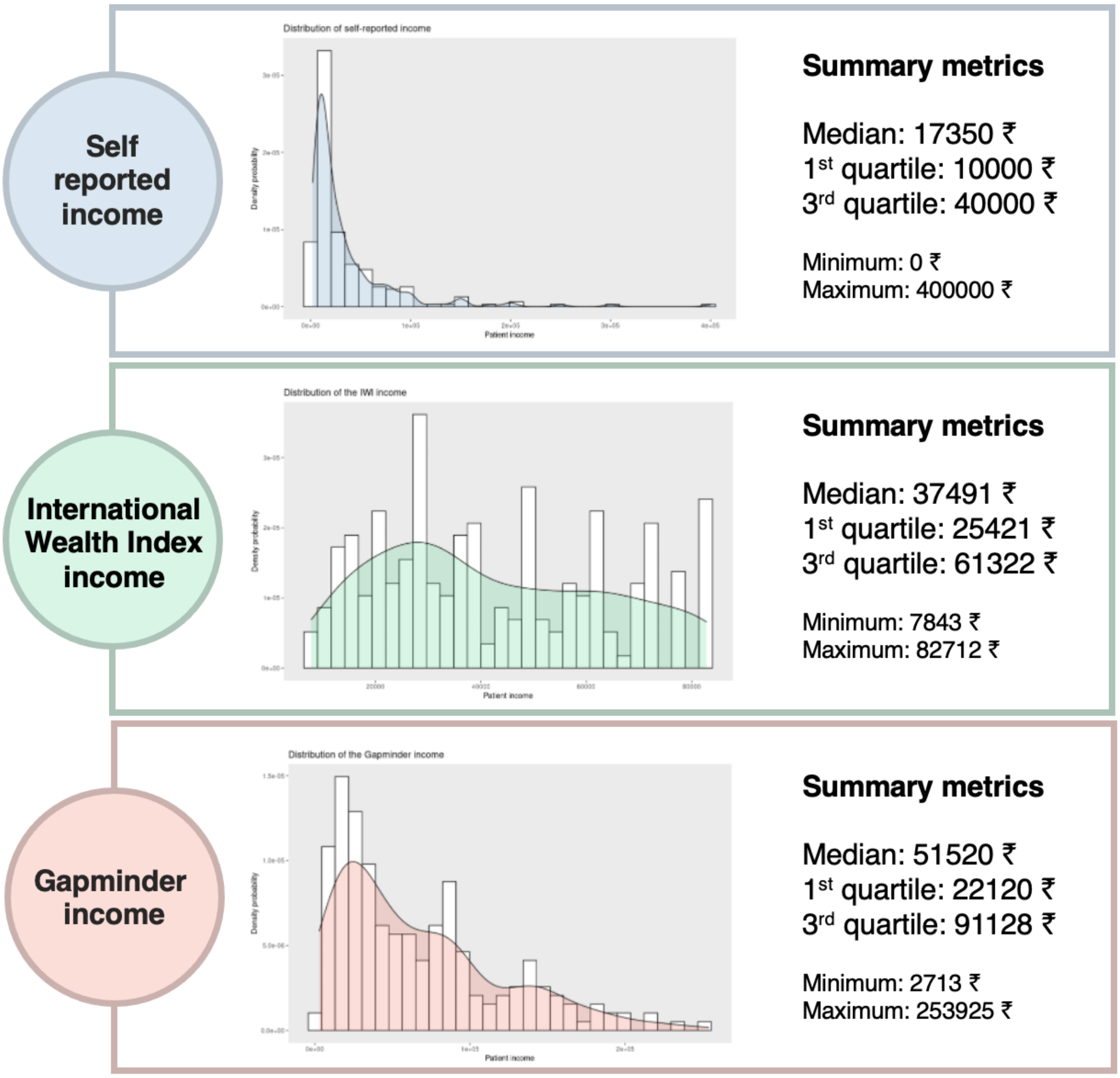

The graphs display histograms of the self-reported, IWI and Gapminder incomes, with overlapped Kernel density plots showing the overall distribution of income figures in the three types of assessment. Summary metrics of the three income assessments are provided to allow comparison. All incomes are reported in Indian Rupees (₹). *Full description of the income methods assessment available in Methods.*

Figure 2: Distribution of all patient household income assessments (self-reported, IWI and Gapminder) in a combined density plot.

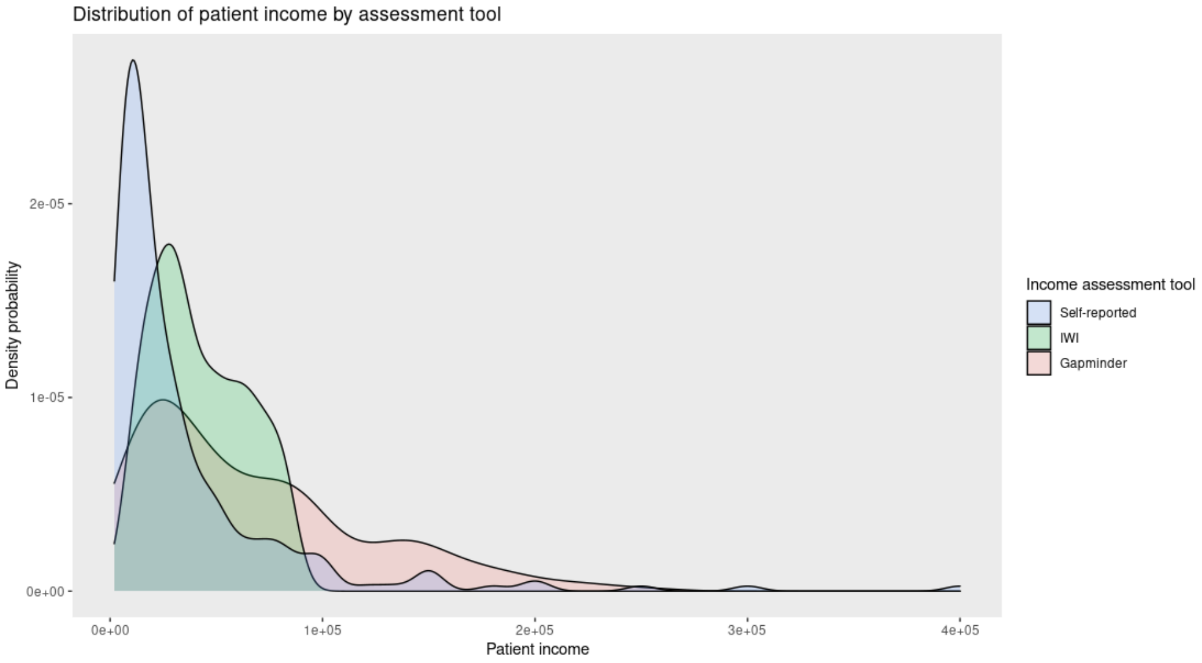

Combined Kernel density plot graph displaying the distribution of incomes assessed by the three income assessment tools (self-reported, IWI and Gapminder tool). Patient income is reported in Indian Rupees (₹).

Figure 3 Click here to access/download;Figure;Figure 3.pdf

Figure 3: Bland-Altman analysis exploring agreement between the self-reported income and the International Wealth Index and the Gapminder incomes.

(A) Bland Altman plots describing the agreement patterns between the self-reported income and the International Wealth Index and the Gapminder incomes, respectively.

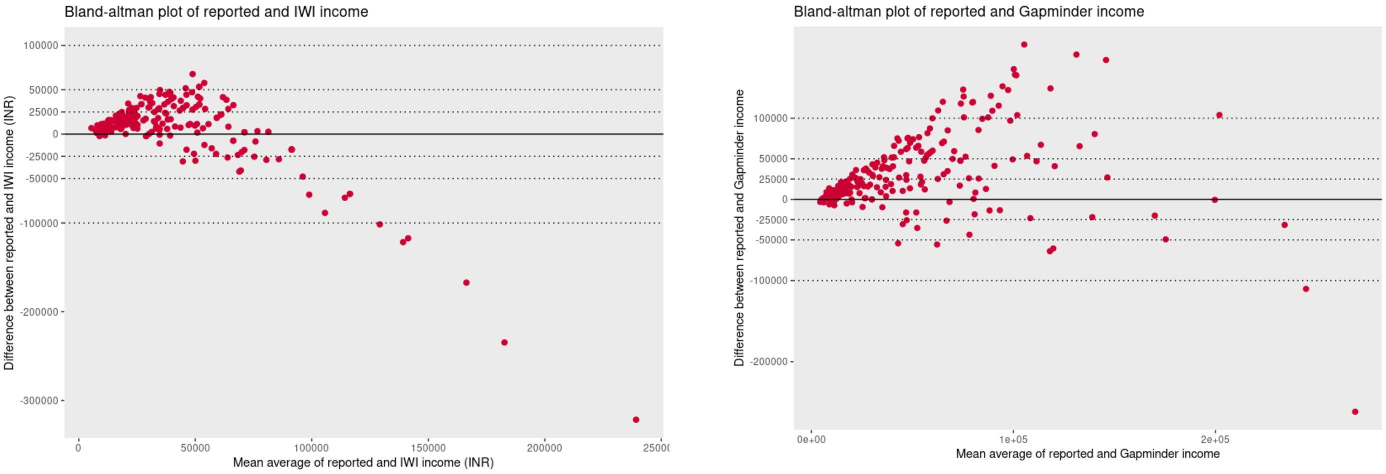

Plots display difference between measured incomes against mean average of measured incomes. The dotted lines represent a difference between income measurements of 25000, 50000 and 100000 INR, respectively. The full line represents perfect agreement (no difference between reported and estimated income).

(B) Proportion of patients for whom the observed difference between the assessed incomes is within 25000, 50000 and 100000 INR.

	Proportion of patients for whom the difference between the assessed incomes is		
	**within a 25000 INR interval**	**within a 50000 INR interval**	**within a 100000 INR interval**
**Reported and IWI income**	67.1% (151/225)	92.8% (209/225)	97.3% (219/225)
**Reported and Gapminder income**	49.3% (111/225)	70.2% (158/225)	88.9% (200/225)

Figure 4: Catastrophic expenditure rates for colorectal cancer care, when using self-reported household income versus the International Wealth Index income or the Gapminder income.

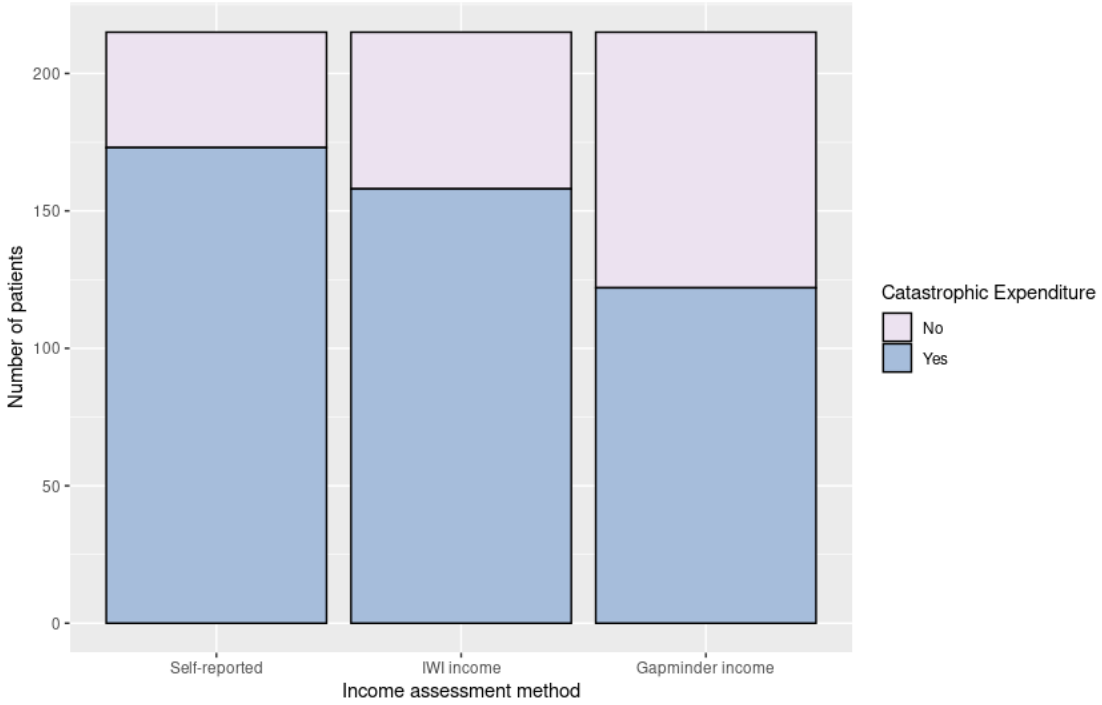

The bar chart compares the number of patients undergoing catastrophic expenditure, when the income is self-reported, assessed through the IWI or the Gapminder tool.

Table 1 Click here to access/download;Figure;Table 1.pdf

Table 1: Demographic and tumour characteristics of the included patients.

Variable	Category	n(%)
Age	18-30 years	18 (8.0)
	31-50 years	83 (36.9)
	51-70 years	96 (42.7)
	>70 years	28 (12.4)
	(Missing)	1
Sex	Male	143 (63.3)
	Female	83 (36.7)
Education level	Primary	51 (22.6)
	Secondary	83 (36.7)
	Graduate	63 (27.9)
	Post-graduate	19 (8.4)
	Did not attend school	10 (4.4)
Job skill level	Low	30 (13.3)
	Medium	40 (17.7)
	High	40 (17.7)
	Not specified - Housewife	65 (28.8)
	Not specified - Retired	39 (17.3)
	Not specified - Other	12 (5.3)
Household size	1-3 people	60 (26.5)
	4-6 people	118 (52.2)
	7-9 people	35 (15.5)
	10+ people	13 (5.8)
Distance from home	0-100 km	77 (34.1)
	100-500 km	73 (32.3)
	500-1000km	10 (4.4)
	>1000 km	66 (29.2)
Hospital type	Government	80 (35.4)
	Charity / Private	146 (64.6)
Cancer location	Colon	108 (47.8)
	Rectum	118 (52.2)
Cancer stage	Local	31 (13.7)
	Advanced	195 (86.3)
Treatment intent	Curative	187 (83.1)
	Palliative	38 (16.9)
	(Missing)	1

Data reported with frequency and proportions as n (%).

Click here to access/download
Supporting Information
Income_Appendix_submission_appendix.docx
